# Evaluating Laparoscopic Sleeve Gastrectomy for Morbid Obesity: A Prospective Follow-Up Study

**DOI:** 10.7759/cureus.61630

**Published:** 2024-06-04

**Authors:** Amar Varshney, Mohammed Fajar Al Sadiq, Mankirat Kaur, Ritu Ramesh Nathawani, Aman Rajadhyaksha, Riya Shailesh Gharat, Kushal G Motwani

**Affiliations:** 1 Department of Surgery, 7 Air Force Hospital, Kanpur, IND; 2 Department of Surgical Gastroenterology, Believers Church Medical College Hospital, Kerala, IND; 3 Department of Surgery, Government Medical College and Hospital, Chandigarh, IND; 4 Department of Obstetrics and Gynecology, Queen's Hospital, London, GBR; 5 Department of Surgery, Goa Medical College and Hospital, Goa, IND; 6 Department of Surgery, Sir Jamsetjee Jeejebhoy Group of Hospital, Grant Government Medical College, Mumbai, IND; 7 Department of Surgery, Sir Byramjee Jeejeebhoy (BJ) Medical College and Civil Hospital, Ahmedabad, IND

**Keywords:** excess weight, weight loss, outcome, morbid obesity, laparoscopic gastric sleeve gastrectomy

## Abstract

Background

Laparoscopic sleeve gastrectomy (LSG) has become a primary option within bariatric surgery (BS), exhibiting favorable outcomes in terms of weight reduction and improvement of associated health conditions. This study was conducted to assess the outcomes of LSG in morbid obesity (MO) in terms of weight reduction and improvement of comorbidities.

Materials and methods

A prospective follow-up study was conducted from January 2021 to January 2023 at the Department of Surgery, 7 Air Force Hospital, Kanpur. The study was approved by the institutional ethical committee with protocol no. IEC/612/2020, including 25 patients diagnosed with MO (BMI >40kg/m^2^) who underwent LSG. Patients were followed up at 1, 3, 6, and 12 months after surgery to track improvements in comorbidities and weight loss. Pre- and post-operative photos were taken, and any complications during the follow-up period were noted.

Results

Most participants in the study were middle-aged individuals, and 84% of the cohort had common comorbidities such as hypertension (HTN) and diabetes mellitus (DM). LSG led to significant and sustained weight loss, with patients achieving an average reduction of 31.56 kg by the 12th month following the surgery. Moreover, substantial improvements in comorbidities, particularly HTN (76.9%) and DM (80%), were observed. However, not all comorbidities exhibited similar rates of recovery, highlighting the need for tailored management strategies. Using a correlation test, no significant correlation was found between the percentage over ideal body weight (IBW) and the reduction in excess weight, as indicated by a p-value exceeding 0.05.

Conclusion

LSG is an effective treatment for severe obesity, delivering significant weight loss and notable improvements in metabolic health and overall quality of life.

## Introduction

Morbid obesity (MO), defined as a condition with a BMI >40kg/m^2^, is a significant global health concern and is associated with numerous debilitating comorbidities and a reduced quality of life [1]. By 2020, it was projected that approximately 30 million adults in India, or about 5% of the adult population, would be obese [2]. Contrary to assumptions, this issue affects both affluent and non-affluent communities across urban and rural landscapes. As non-surgical treatments have their limitations, bariatric surgery (BS) has become an important therapeutic option, especially for those with severe obesity (BMI > 40 kg/m2) [3-5].

Laparoscopic sleeve gastrectomy (LSG) has become increasingly popular among bariatric procedures due to its positive results in sustainable weight loss and improving obesity-related comorbidities [6, 7]. LSG and laparoscopic Roux-en-Y gastric bypass (LRYGP) are the primary choices for BS in India [8]. LSG has garnered attention for its effectiveness in achieving weight loss, with patients typically experiencing a reduction of 50% to 70% of excess weight within the initial postoperative year [9]. Furthermore, LSG shows beneficial outcomes in addressing obesity-related comorbidities like type 2 diabetes and hypertension, while also presenting a lower risk profile compared to alternative surgical methods [10].

The surge in obesity prevalence underscores the imperative for a rigorous evaluation of BS to inform evidence-based clinical practice. A prospective follow-up study offers the optimal method for examining the long-lasting efficacy and safety profile of LSG in the treatment of MO. By systematically tracking patient outcomes over an extended period, this study aims to provide comprehensive insights into weight loss, metabolic improvements, and postoperative complications associated with LSG. These longitudinal evaluations allow healthcare professionals to analyze the enduring effects of LSG on maintaining weight loss, resolving metabolic syndrome, and enhancing overall patient welfare. Furthermore, the inclusion of a prospective cohort facilitates the exploration of potential predictors of surgical success and the identification of patient subgroups most likely to benefit from LSG.

This study aims to detail the characteristics and outcomes of patients diagnosed with both MO and metabolic syndrome who underwent LSG at a tertiary care hospital in India. Through a comparative analysis of available literature, our goal is to advance understanding regarding the effectiveness and relevance of LSG in addressing the complex interaction between obesity and metabolic disorders.

## Materials and methods

A prospective follow-up design was utilized in this study to evaluate laparoscopic sleeve gastrectomy (LSG) outcomes in morbidly obese patients. This study was conducted from January 2021 to January 2023 at the Department of Surgery, 7 Air Force Hospital, Kanpur, and the research proposal was approved by the Institutional Ethics Committee of the Surgery Department at 7 Air Force Hospital, Kanpur, with protocol number IEC/612/2020. Subjects aged 18 to 70 years, who met the WHO guidelines for obesity surgery and expressed willingness for LSG, were enrolled. Only patients who had unsuccessfully attempted conservative management were considered. Individuals below 18 or above 70 years of age were excluded. Since the study focuses on a very specific population subgroup, a small sample size was inevitable due to the limited pool of eligible participants. A confidence level of 95% with a margin of error of 5% and a total population size of 25 were required. After rigorous screening, 25 highly motivated patients were selected. Before the surgery, patients were provided comprehensive counseling regarding LSG specifics, associated risks, and potential postoperative complications.

Detailed preoperative assessments were conducted, including patient history, duration of weight gain, associated comorbidities, previous weight loss attempts, medical therapy history, general physical examination, weight record, and optimization of various physiological systems such as endocrine, cardiology, pulmonary, and psychiatry. Additionally, patients underwent specific investigations, including an echocardiogram, pulmonary function test (PFT), serum insulin, serum cortisol (to rule out Cushing's syndrome), and ultrasound (to confirm or exclude gallstone disease). Patients diagnosed with gallstone disease underwent laparoscopic cholecystectomy (LC) during LSG. Spirometry incentives were initiated preoperatively, and patients were advised to adhere to a fluid diet for one week before the procedure.

Under general anesthesia, laparoscopic gastric sleeve gastrectomy (LSG) was carried out using four ports. The patient was placed in a steep "anti-Trendelenburg" position with legs separated, while the surgeon was positioned between the patient's legs. Additional ports were added if multiple LC operations were required simultaneously. Postoperatively, patients wore pneumatic compression stockings, received antibiotics during induction, and received oral fluids on the evening of surgery. Detailed dietary instructions were provided upon discharge on the fourth postoperative day. BS group's weight loss progress was measured using the percentage change in BMI and the percentage of excess weight loss. The percentage change in BMI was calculated as 100% × ([initial BMI-current BMI]/initial BMI), where the initial BMI (BMI0) is the BMI at the time of surgery and the current BMI (BMI) is the BMI at the latest follow-up. The percentage of excess weight loss was determined by 100% × ([initial weight - current weight]/excess weight at the time of surgery), where the initial weight (W0) represents the weight at the time of surgery, the current weight (Wi) represents the weight at the latest follow-up, and the excess weight (EW0) represents the excess weight at the time of surgery. Excess weight was estimated using the formula described by Devine BJ [11].

Follow-up visits were scheduled for participants at 1, 3, 6, and 12 months post-surgery. Documentation included tracking improvements in comorbidities and weight loss and capturing pre- and post-operative images. Any complications during the follow-up period were also recorded. Statistical analysis was carried out using SPSS version 21, employing the paired t-test with a significance level of p ≤ 0.05.

## Results

The baseline features of the study participants are summarized in Table 1. Variables included gender distribution, age groups, the presence of comorbidities, and associated diseases. The data are presented as counts and percentages within each category. Out of the total 25 patients who underwent LSG during the study, 5 (20.0%) were male and 20 (80.0%) were female. Participants were divided into different age categories, with the majority (40%) being in the age group of 41-50 years. Among the participants, 4 (16.0%) had comorbidities, while 21 (84.0%) had no comorbid conditions. The study also examined various associated diseases among the participants; hypertension (HTN) was present in the majority (13 or 52.0%), followed by diabetes in 10 (40.0%) and skin infection in 6 (24.0%).

**Table 1:** Baseline variables of the study participants. PCOD: Polycystic ovarian disease; GERD: Gastroesophageal reflux disease; DVT: Deep vein thrombosis.

S. No.	Variables		N (%)
1.	Gender	Male	5 (20.0%)
		Female	20 (80.0%)
2.	Age groups	18-20	1 (4.0)
		21-30	3 (12.0)
		31-40	6 (24.0)
		41-50	10 (40.0)
		51-60	5 (20.0)
3.	Comorbidities	Absent	4 (16.0)
		Present	21 (84.0)
4.	Associated diseases	Hypertension	13 (52.0)
		Diabetes	10 (40.0)
		Skin infection	6 (24.0)
		Osteoarthritis	5 (20.0)
		Hypothyroidism	5 (20.0)
		PCOD	3 (12.0)
		GERD	1 (4.0)
		DVT	1 (4.0)
5.	BMI kg/m^2^	35-39.9	6 (28.0)
		>40	18 (72.0)

The weight loss progression of individuals who underwent BS over the course of one year is detailed in Table 2. The data were presented at various time intervals following the surgery, starting from baseline and progressing to the 3rd, 6th, 9th, and 12th months. The participants exhibited a statistically significant decrease in weight at each subsequent time point compared to baseline, evidenced by the declining mean weight and p-values below 0.05. The most substantial weight loss was observed by the 12th month after surgery, with an average reduction of 31.56 kg compared to baseline.

**Table 2:** Weight loss in bariatric surgery patients. P-value <0.05 was considered statistically significant.

Duration	Number of patients	Weight in kg (mean±SD)	P-value
At baseline	25	113.96±24.13	-
3rd month	25	101.80±21.26	0.021
6th month	25	93.44±18.03	<0.001
9th month	25	86.60±15.43	<0.001
12th month	25	82.40±14.83	<0.001

Table 3 presents the postoperative outcomes of study participants with various comorbidities one year after undergoing surgery. The outcomes are categorized into three groups: complete recovery, partial recovery, and no change. For comorbidities such as skin infection, hypothyroidism, and polycystic ovary syndrome (PCOS), all participants experienced complete recovery (100%) after 12 months. In conditions like GERD and DVT, there was either no improvement or complete absence of recovery observed in the participants. For HTN and diabetes mellitus (DM), a significant proportion of study participants experienced complete or partial recovery, indicating the positive impact of the surgery on these conditions.

**Table 3:** Post-operative outcomes after a 12-month follow-up. HTN: Hypertension; DM: Diabetes Mellitus; OA: Osteoarthritis; PCOD: Polycystic ovarian disease; GERD: Gastroesophageal reflux disease; DVT: Deep vein thrombosis.

Comorbidities	Number of patients	Complete recovery (% change)	Partial recovery (% change)	No change (% change)
HTN	13	8 (61.5)	2 (15.4)	3 (23.1)
DM	10	4 (40.0)	4 (40.0)	2 (20.0)
Skin infection	6	6 (100.0)	0 (0.0)	0 (0.0)
OA	5	3 (60.0)	2 (40.0)	0 (0.0)
Hypothyroidism	5	5 (100.0)	0 (0.0)	0 (0.0)
PCOD	3	3 (100.0)	0 (0.0)	0 (0.0)
GERD	1	0 (0.0)	1 (100.0)	0 (0.0)
DVT	1	0 (0.0)	0 (0.0)	1 (100.0)

**Figure 1:**
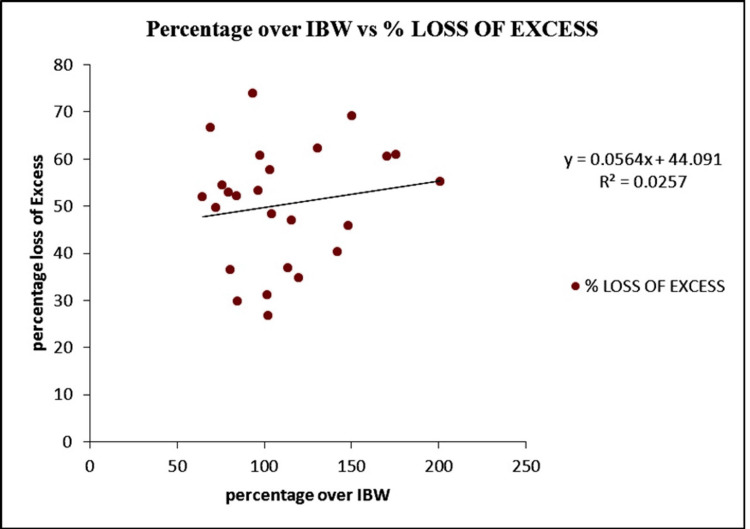
The correlation between percentage over IBW and percentage loss of excess weight. IBW: Ideal body weight.

Figure 1 shows the correlation between percentage over ideal body weight (IBW) and percentage loss of excess weight. Using a correlation test, no significant correlation was identified between the percentage over IBW and the percentage of excess weight loss.

## Discussion

The findings of this prospective follow-up study highlight the efficacy and outcomes of LSG in managing MO and its associated comorbidities. The distribution of age groups shows that middle-aged individuals, particularly those aged 41-50 years, comprised a significant portion of the study population. This demographic profile is typical of BS candidates, who often experience a peak in obesity-related health issues during middle age [12, 13].

The prevalence of comorbidities and associated diseases among the study cohort underscores the multifaceted nature of morbid obesity and its impact on overall health. HTN and DM were the most prevalent comorbidities, consistent with previous research demonstrating the strong association between obesity and these conditions. Similarly, Shuvo SD et al. reported a comparable prevalence of comorbid conditions among obese patients [14]. Other associated diseases, such as skin infections, osteoarthritis, and hypothyroidism, further highlight the diverse health challenges faced by individuals with morbid obesity [15, 16]. The significant reduction in mean weight at each subsequent time point reflects the effectiveness of LSG in achieving sustainable weight loss outcomes. These findings are consistent with existing literature demonstrating the superior efficacy of LSG in promoting significant weight reduction compared to non-surgical interventions [17-20].

The significant decrease in weight noted as soon as the 3rd month after surgery emphasizes the swift and substantial influence of LSG on weight loss. By the 12th month, patients witnessed a noteworthy average weight reduction, underscoring the enduring efficacy of LSG in promoting weight loss maintenance. These results reaffirm LSG's status as a preferred bariatric procedure for achieving considerable and lasting weight loss outcomes in individuals with MO [21, 22]. The significant rates of full recovery for comorbidities such as HTN, DM, and skin infections underscore the extensive metabolic and health benefits of LSG beyond simple weight loss. These results align with prior studies showing the positive effects of LSG on resolving comorbidities and enhancing metabolic indicators [23, 24].

However, it is noteworthy that not all comorbidities exhibited similar rates of recovery, with conditions like GERD and DVT showing limited or no improvement postoperatively. This underscores the need for continued monitoring and tailored management of comorbidities among BS participants, particularly those with complex medical histories. The absence of a significant correlation between percentage over IBW and percentage loss of excess weight indicates that factors beyond initial excess weight may influence the magnitude of weight loss following LSG. This highlights the importance of individualized subject assessment and consideration of various clinical factors in predicting postoperative outcomes.

Limitations

The study's small sample size may limit the generalizability of the findings to a broader population. A larger sample size would yield more robust results and allow for enhanced statistical analysis. Longer follow-up periods are needed to assess the sustainability of weight loss and the persistence of metabolic improvements over time.

## Conclusions

Overall, the findings from this study provide significant insights into the efficacy and consequences of LSG in managing MO and its accompanying comorbidities. This underscores LSG's role as a safe and effective therapeutic option for individuals with severe obesity, offering substantial weight loss and notable improvements in both metabolic health and overall well-being. These results hold crucial implications for clinical practice, stressing the importance of multidisciplinary care and customized interventions to optimize outcomes for patients undergoing bariatric surgery.
